# Supplementary material for: Small-Medium Extracellular Vesicles and Their miRNA Cargo in Retinal Health and Degeneration: Mediators of Homeostasis, and Vehicles for Targeted Gene Therapy
Source: Front Cell Neurosci. 2020 Jun 25;14:160. doi: 10.3389/fncel.2020.00160 (PMC7330137; doi:10.3389/fncel.2020.00160)
Supplement: Supplementary file 2 [file Presentation_1.pdf]

1 Supplementary Figures

A Retinal s-mEV

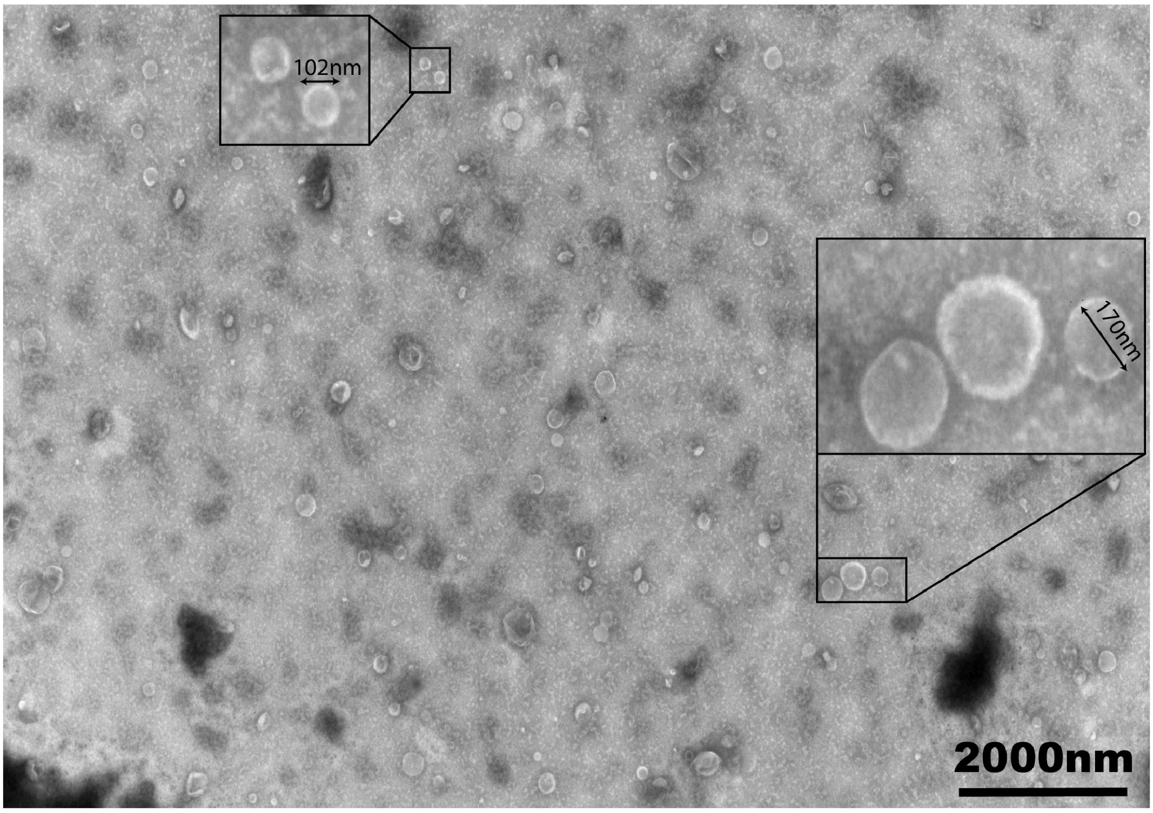

B 661W s-mEV

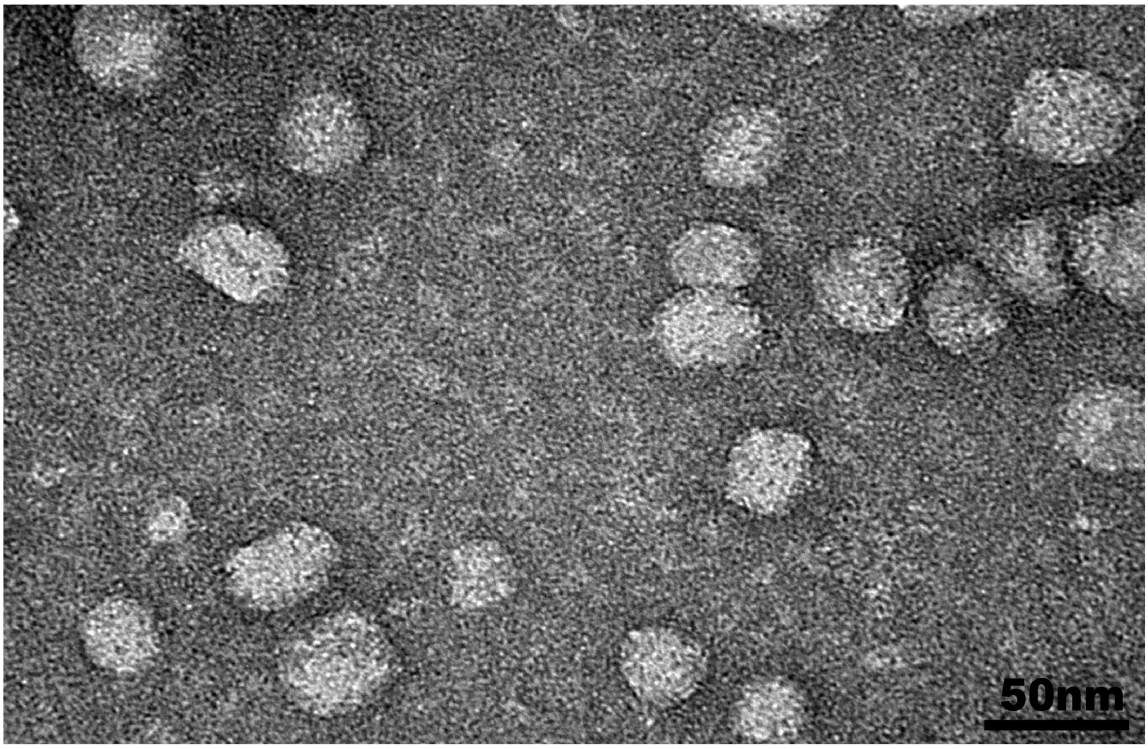

Figure S1. Representative TEM image showing isolated extracellular vesicles from **(A)** retinas and **(B)** 661W cells. Enlarged fields highlight the circular, cup-shape morphology and size of s-mEV such as exosomes. Scale bar = 2000nm and 50nm.

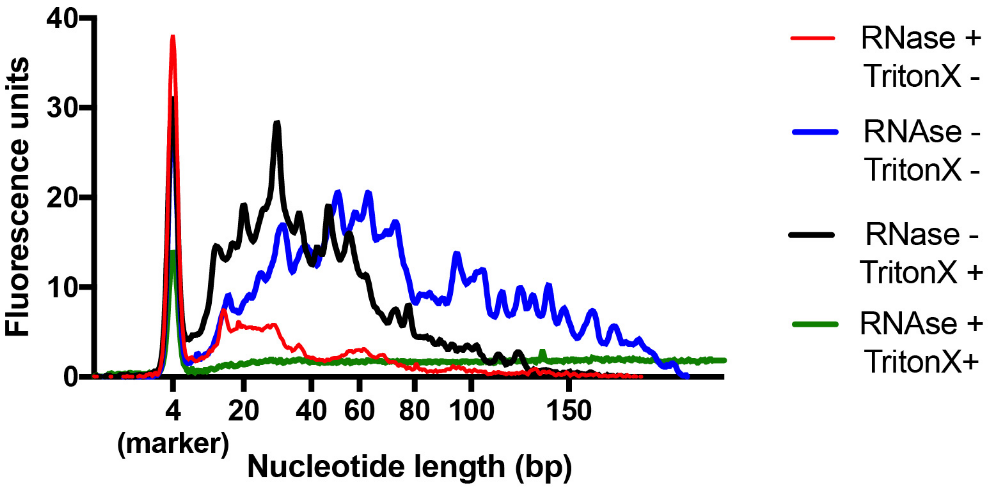

Figure S2. RNaseA treatment.

s-mEV were treated with RNaseA with or without Triton-X 100 for 30 mins then processed immediately for RNA extraction. Exosomes treated with RNAaseA without Triton-X 100 (red line) showed a decrease in the total amount of RNA compared with untreated s-mEV (blue line), and total RNA (black line), indicating the presence of contaminating RNA. The RNA protected from RNaseA degradation (red line) was enriched for RNA species of 10 to 40 nucleotides (bp) in length. The simultaneous treatment with RNasA and Triton-X 100 (green line) completely ablated the signal, further demonstrating that s-mEV-RNA is protected from RNAseA degradation.

Read trimming, alignment and normalization

A Sequencing reads summary

Sample name	Total reads	Read length	Avg. read quality	% N	% GC
DR1	14,577,486	51.00	33.13	0%	43.77%
DR2	13,972,828	51.00	33.19	0%	44.37%
DR3	15,003,428	51.00	33.19	0%	44.72%
DR4	8,960,555	51.00	32.43	0%	41.65%
DR5	10,009,182	51.00	32.64	0%	42.05%
DR6	9,918,357	51.00	32.37	0%	42.67%
PD1	15,073,063	51.00	33.24	0%	45.01%
PD2	15,641,433	51.00	33.20	0%	45.43%
PD3	11,058,946	51.00	33.13	0%	44.18%
PD4	11,413,904	51.00	32.55	0%	41.06%
PD5	8,104,058	51.00	32.20	0%	42.43%

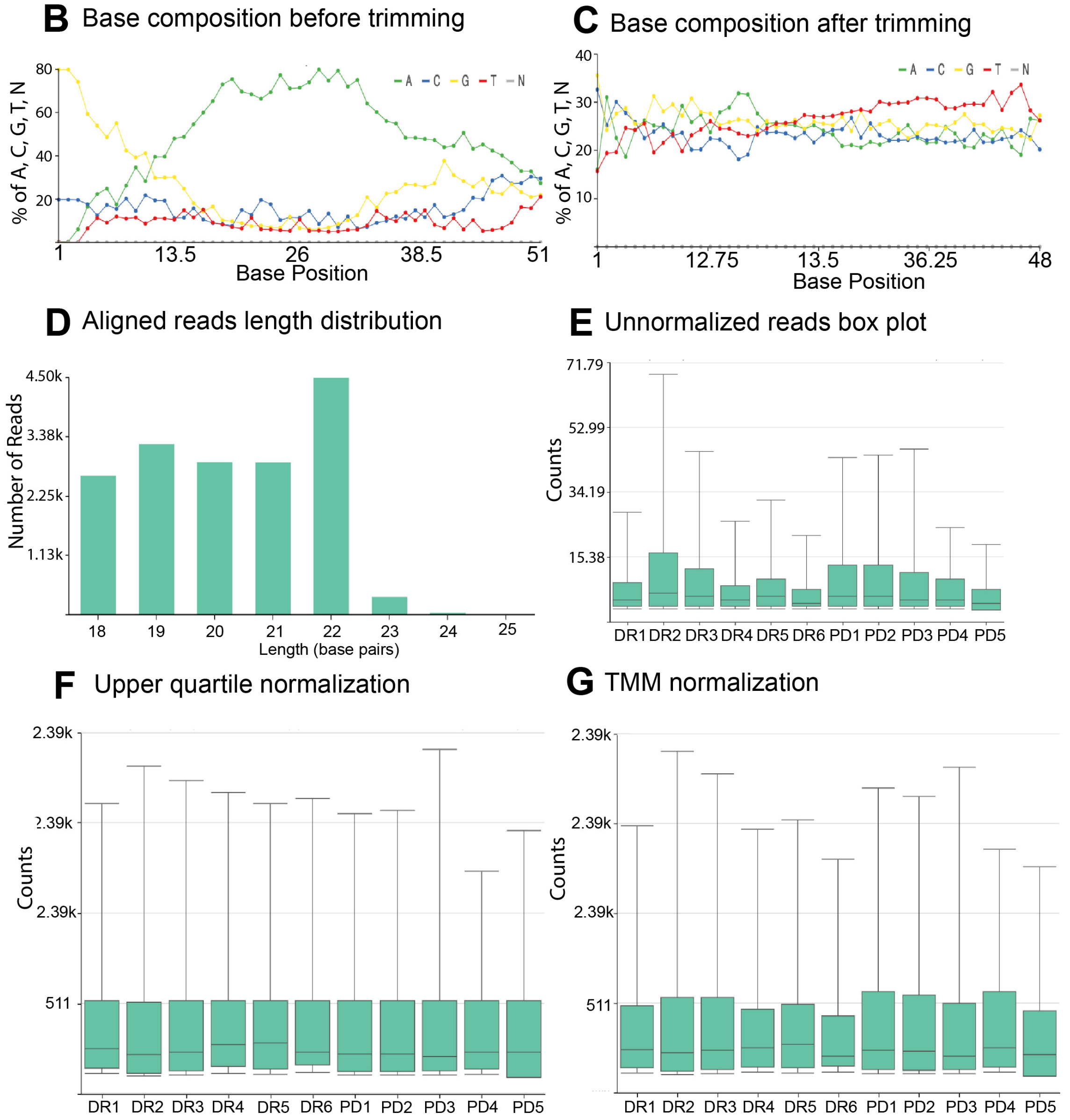

Figure S3. Sequenced reads processing, alignment and normalization. (A) Summary of the sequencing depth, read quality and GC content. **(B)** Representative raw read base composition shows the presence of an enriched poly-A tail (green line) and template switching nucleotide (first 3 base pairs). These features are incorporated during the CATS library preparation and thus their enrichment in the sequenced reads indicates the successful construction of the library.

(C) After trimming, every nucleotide was equally represented along the sequenced reads indicating the successful removal of library contaminants. **(D)** Reads aligning to miRbase v.22 (mature miRNA) had length distribution ranging from 18 to 24 nucleotides consistent with the length of annotated miRNAs. **(E)** After annotation, unnormalized counts were normalized using the **(F)** upper quartile (UQ) normalization or **(G)** Trimmed Means of M, with the former producing more similar means and distributions (each box-and whisker plot corresponds to a sample).
